# Polyadenylation Linked to Transcription Termination Directs the Processing of snoRNA Precursors in Yeast

**DOI:** 10.1016/j.molcel.2008.10.003

**Published:** 2008-10-24

**Authors:** Pawel Grzechnik, Joanna Kufel

**Affiliations:** 1 Institute of Genetics and Biotechnology, Faculty of Biology, University of Warsaw, Pawinskiego 5a, 02-106 Warsaw, Poland

**Keywords:** RNA

## Abstract

Transcription termination by RNA polymerase II is coupled to transcript 3′ end formation. A large cleavage and polyadenylation complex containing the major poly(A) polymerase Pap1 produces mRNA 3′ ends, whereas those of nonpolyadenylated snoRNAs in yeast are formed either by endonucleolytic cleavage or by termination, followed by trimming by the nuclear exosome. We show that synthesis of independently transcribed snoRNAs involves default polyadenylation of two classes of precursors derived from termination at a main Nrd1/Nab3-dependent site or a “fail-safe” mRNA-like signal. Poly(A) tails are added by Pap1 to both forms, whereas the alternative poly(A) polymerase Tfr4 adenylates major precursors and processing intermediates to facilitate further polyadenylation by Pap1 and maturation by the exosome/Rrp6. A more important role of Trf4/TRAMP, however, is to enhance Nrd1 association with snoRNA genes. We propose a model in which polyadenylation of pre-snoRNAs is a key event linking their transcription termination, 3′ end processing, and degradation.

## Introduction

Synthesis of mature RNAs in eukaryotic cells is a multistep process. Accumulation and function of small nucleolar RNAs (snoRNAs) require correct transcription termination, 3′ and 5′ processing, and assembly into ribonucleoprotein particles (RNPs). In *Saccharomyces cerevisiae*, two classes of snoRNAs, box C/D and box H/ACA, act in preribosomal RNA processing and modification. They have distinct secondary structures, associate with different sets of proteins, and guide unrelated modification reactions (2′-O-ribose methylation versus pseudouridylation) (Kiss, 2002). They are synthesized as larger precursors by RNA polymerase II (Pol II) from independent transcription units, polycistronic precursors, or excised introns. Following transcription termination, cleavage by endonuclease Rnt1 (RNase III) or release from introns, their mature 3′ ends are generated by the exosome, a complex with the 3′→5′ exonuclease activity (Allmang et al., 1999; van Hoof et al., 2000). Transcription termination of snoRNAs involves a complex of two RNA-binding proteins, Nrd1 and Nab3, and a putative RNA helicase, Sen1. Nrd1 interacts with the C-terminal domain (CTD) of Pol II and with the exosome to link termination with processing (Kim et al., 2006; Steinmetz et al., 2001; Vasiljeva and Buratowski, 2006). Some components of the mRNA 3′ end formation apparatus, including subunits of the mRNA cleavage factor IA (Rna14, Rna15, Pcf11), subunits of the Pti1-associated cleavage and polyadenylation factor (CPF) subcomplex APT (Ref2, Ssu72, Pta1, Swd2, Glc7), and the PAF complex, also contribute to snoRNA termination (Cheng et al., 2004; Dheur et al., 2003; Dichtl et al., 2004; Fatica et al., 2000; Ganem et al., 2003; Morlando et al., 2001; Nedea et al., 2003, 2008; Sheldon et al., 2005; Steinmetz and Brow, 2003). This process has been proposed to occur in a cleavage-dependent manner (Fatica et al., 2000; Morlando et al., 2001), but the potential nuclease has not been identified. CPF component Brr5, a yeast homolog of the mRNA 3′ end-processing endonuclease, was shown not to be involved. Nucleases, such as Rnt1, the exosome, and particularly Rat1 that participates in termination of mRNAs, are also not important for snoRNA termination (Kim et al., 2006).

A bipartite signal directing termination of snoRNA transcripts was identified for snR13, snR50, snR65, and snR47. It consists of a Nrd1/Nab3-binding region (site I) and a sequence resembling the mRNA cleavage/polyadenylation signal (site II) (Fatica et al., 2000; Morlando et al., 2001; Steinmetz and Brow, 2003; Steinmetz et al., 2006). Mutations in these elements, as in *trans*-acting factors, lead to transcriptional readthrough of the terminators. Efficient termination requires cotranscriptional recruitment of Nrd1/Nab3 and cleavage and polyadenylation components to snoRNA genes. In addition, the Nrd1/Nab3-dependent pathway involves interaction between Nrd1 and the phosphorylated Ser-5 of CTD and direct binding of several Nrd1 and Nab3 molecules to multiple sites in the RNA (Gudipati et al., 2008; Kim et al., 2006; Steinmetz and Brow, 2003; Vasiljeva et al., 2008b).

Nuclear exosome, and above all its nuclear component Rrp6 and cofactor Rrp47, has long been known to participate in the formation of snoRNA 3′ ends. The absence of exosomal subunits leads to accumulation of 3′ unprocessed forms, carrying either a few additional residues or longer polyadenylated extensions (Allmang et al., 1999; LaCava et al., 2005; Mitchell et al., 2003; van Hoof et al., 2000). Although some polyadenylation of pre-snoRNAs by the classical mRNA poly(A) polymerase Pap1 has been reported (van Hoof et al., 2000; Wyers et al., 2005), major activities accountable for addition of these poly(A) tails are the highly related poly(A) polymerases Trf4 and Trf5 (Egecioglu et al., 2006; Houseley and Tollervey, 2006; LaCava et al., 2005; Vanacova et al., 2005; Wyers et al., 2005). Together with RNA-binding proteins Air1/2 and an exosome cofactor, DEVH ATP-dependent RNA helicase Mtr4, they form TRAMP complexes, which activate the nuclear exosome and polyadenylate a large number of defective, superfluous, or regulated RNAs such as unmodified tRNAs, unprocessed rRNAs, and cryptic unstable transcripts (CUTs) (Davis and Ares, 2006; Dez et al., 2006; Houseley et al., 2007; Kadaba et al., 2004, 2006; Wyers et al., 2005). Trf4/5-mediated polyadenylation is believed to mark the bulk of RNAs for degradation and thus acts as a nuclear surveillance mechanism.

Although polyadenylation of intrinsically nonpolyadenylated sn/snoRNAs was attributed to the RNA quality control, polyadenylation-driven processing of precursors cleaved by Rnt1 has also been suggested (Egecioglu et al., 2006; van Hoof et al., 2000). We have therefore investigated the relationship between snoRNA 3′ end formation and polyadenylation and the extent of Pap1 and Trf4/5 contribution to this process. We find that 3′ ends unprocessed in the absence of Rrp6 contain uncoded adenines that probably represent vestiges of the initial poly(A) tail. Our experiments show that snoRNA termination at both sites is followed by polyadenylation by Pap1 that initiates 3′ end processing by the nuclear exosome/Rrp6. Trf4 contributes to the association of Nrd1 with snoRNA genes and adenylates pre-snoRNAs to facilitate their processing. During the final steps of maturation, snoRNA fate is decided, and incorrectly processed or misassembled molecules are degraded by TRAMP and the exosome.

## Results

### 3′ Ends of Box C/D snoRNAs Are Oligoadenylated in the Absence of Rrp6

**Figure 1:**
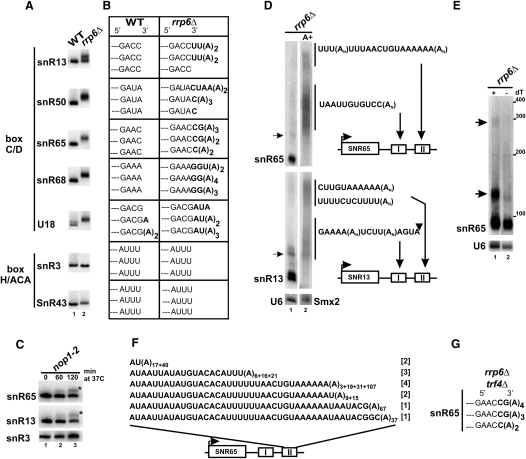
Polyadenylated snoRNA Species in the *rrp6Δ* Strain Originate from Transcription Terminators (A, B, and G) SnoRNAs are oligoadenylated in the absence of Rrp6. Northern hybridization and CR-RT-PCR analysis of different box C/D and box H/ACA snoRNAs in wild-type and *rrp6Δ* strains (A and B). CR-RT-PCR for snR65 in the *rrp6Δ/trf4Δ* strain (G). Only the last few residues of mature ends are shown; undigested nucleotides and adenines are in bold. (C) Inactivation of Nop1 in a temperature-sensitive *nop1-2* strain leads to accumulation of adenylated boxC/D snR13 and snR65 (marked with asterisks). (D) Northern hybridization of total and poly(A)^+^ RNA from the *rrp6Δ* strain for snR65 and snR13. U6 and *SMX2* mRNA are loading controls for total and poly(A)^+^ RNAs, respectively. Two poly(A)^+^ populations are indicated by vertical lines. Arrows show major oligoadenylated precursor terminated at site I. Sequencing of RT-PCR products on polyadenylated precursors from the *rrp6Δ* strain is shown on the right. An arrowhead indicates the cleavage site in terminator I of snR13. (E) RNase H treatment of total RNA from the *rrp6Δ* strain in the presence of oligo(dT) for snR65. Arrows point at deadenylated species. RNA size marker is on the right. (F) CR-RT-PCR of polyadenylated pre-snR65 terminated in region II in the *rrp6Δ* strain. The number of added A residues is in subscript, and numerals in parentheses denote number of clones.

The last step of 3′ end processing of independently transcribed and intron-encoded snoRNAs involves trimming by the nuclear exosome component, Rrp6, with the cooperation of Rrp47. In their absence, box C/D snoRNAs contain short 3′ extensions that have been assumed to represent the last undigested nucleotides of the precursor (Allmang et al., 1999; Mitchell et al., 2003; van Hoof et al., 2000). We have re-examined 3′ ends of box C/D snoRNAs snR13, snR50, snR65, snR68, and U18 in the *rrp6Δ* strain. Circular RT-PCR (CR-RT-PCR) followed by sequencing shows that they carry one to six additional residues, of which only the first few overlap with those in the precursor, the remaining ones being one to four adenosines not present in the sequence (Figures 1A and 1B). In contrast, in the wild-type strain only mature 3′ ends are reproduced for all snoRNAs tested except intron-encoded U18. A similar analysis for box H/ACA snoRNAs snR3 and snR43 reveals the same mature 3′ termini in both strains. The untemplated adenosines at 3′ ends of box C/D snoRNAs in the *rrp6Δ* strain probably represent incompletely processed precursors. As 3′ extended and adenylated sn/snoRNAs are stable and functional (Abou Elela and Ares, 1998; van Hoof et al., 2000), it raises the possibility that snoRNA processing may involve addition of 3′ poly(A) tails.

3′ extended species were also observed for intron-encoded box C/D snoRNAs in mutants in a core snoRNP protein, Nop1 (Lafontaine and Tollervey, 2000). We have tested 3′ ends of snR65 and snR13 in the *nop1-2* temperature-sensitive mutant (Figure 1C). Inactivation of Nop1 results in a gradual depletion of mature snoRNAs, as reported, and accumulation of short heterogeneous species. They correspond to oligoadenylated snoRNAs that probably failed to be properly assembled into the RNP and, as a result, became inaccurately matured by Rrp6 and eventually degraded. This result points to the correlation between final processing steps and snoRNP assembly.

### Polyadenylated snoRNAs in the *rrp6Δ* Strain Are Precursor Species

Longer polyadenylated snoRNA forms that accumulate in the *rrp6Δ* strain were examined by northern analysis of the poly(A)^+^ fraction selected on an oligo(dT) column. Two poly(A)^+^ populations were detected for box C/D snR65, snR13, snR64, snR68, and U14 as well as box H/ACA snR46 and snR33 (Figure 1D and see Figure S1 available online). The shorter set starts at the height of the primary precursor (shown with an arrow in Figure 1D). Both poly(A) tails span approximately 70–80 nt as estimated by comparison of the untreated and deadenylated snR65 by RNase H in the presence of oligo(dT) (Figure 1E). In the deadenylated sample, two polyadenylation sites are visible, located ∼30 and 180 nt from the mature 3′ end, which agrees well with the positions of the two terminators. Assessment of poly(A) sites for snR65 and snR13 precursors by RT-PCR against the poly(A) tail, followed by sequencing, shows that they are located in terminator I and II (Figure 1D). In wild-type cells, polyadenylated species were mapped to terminate at the same positions in region I (data not shown). One of the sites in snR13 lies 4 nt upstream of the potential cleavage (Morlando et al., 2001). An additional mRNA-like element, which conforms well to the Py(A)*n* consensus, is present in snR13 downstream of known region II. The exact location of poly(A) sites and the number of added adenines in terminator II of pre-snR65 were determined by CR-RT-PCR. Of 13 sequenced clones, all contain from 3 to 107 As at different positions within this region (Figure 1F). For technical reasons, it was difficult to apply this approach to RNAs derived from site I.

These data show that snoRNA species are not polyadenylated at random sites but at two discrete regions corresponding to terminators I and II. We predict that they do not represent stabilized exosome degradation intermediates but snoRNA precursors that are generated by polyadenylation linked to transcription.

**Figure 2:**
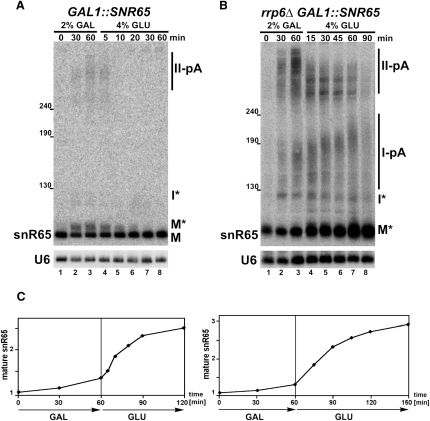
SnoRNAs Are Synthesized from Two Classes of Polyadenylated Precursors Transcriptional pulse stop of snR65 in *GAL1::SNR65* (A) and *GAL1::SNR65*/*rrp6Δ* (B) strains. Transcription was induced for 60 min and then inhibited. I-pA and II-pA, polyadenylated precursors from respective termination sites; I^∗^, oligoadenylated precursor from site I; M^∗^, semimature species; M, mature snoRNA. (C) PhosphorImager quantification of data from (A) and (B) for mature snR65. Values are standardized to the U6 control and expressed relative to levels before induction.

To test this possibility, transcriptional pulse stop induced by galactose and stopped by glucose was performed for snR65 expressed from an inducible *GAL1* promoter (*GAL1::SNR65*) (Figure 2A). Owing to a certain leakage from the *GAL1* promoter, there is a background level of snR65 before the pulse. Two classes of newly synthesized pre-snR65, with significantly different sizes (130 nt for species labeled I^∗^ and 280–340 nt for species labeled II-pA) are visible after 30 min following the pulse. The heterogeneous II-pA is polyadenylated, as attested by deadenylation with RNase H and oligo(dT) (data not shown), and matches precursors resulting from termination in region II. The shorter RNA, which resembles species in *rrp6Δ* cells shown with arrows in Figure 1D, does not carry a poly(A) tail but may well be oligoadenylated, as some diffused forms are visible. A more abundant third species, marked as M^∗^, also accumulates, only a few nucleotides longer than the mature snR65. It represents semimature snoRNA that is normally trimmed by Rrp6 and is predominant in its absence (see Figure 1A). Accumulation of two polyadenylated heterogeneous pre-snR65, I-pA and II-pA, and of the adenylated I^∗^ species is much stronger in the *rrp6Δ* mutant (Figure 2B). I-pA is not seen in the wild-type by northern analysis, but both polyadenylated forms are detected using RT-PCR against the poly(A) tail (Figure S2), showing that in wild-type and *rrp6Δ* cells I-pA and I^∗^ correspond to major precursors generated at terminator I. Following transcription inhibition, all precursors and intermediates in the wild-type (II-pA, I^∗^ and M^∗^) and II-pA RNAs in the *rrp6Δ* are shortened and disappear with slightly different kinetics. This is accompanied by the buildup of the mature or semimature (M^∗^) snoRNAs in wild-type or *rrp6Δ* cells, respectively (Figure 2C, see also Figure S3), suggesting that they arise from the processing of these precursors. Surprisingly, I^∗^ and I-pA persist, and I-pA becomes hyperadenylated in the absence of Rrp6. These species are probably not converted to mature RNAs, showing that their processing or decay strictly depends on Rrp6. Different behavior of I-pA and II-pA suggests that they are produced as separate precursors, while I^∗^ and I-pA are interrelated.

Assessment of the transcript kinetics allows us to conclude that snoRNAs are processed from polyadenylated precursors by independent termination at site I or II, immediately followed, either co- or posttranscriptionally, by the addition of poly(A) tails at both terminators. In wild-type cells, snoRNAs are generated from either precursor but preferably from polyadenylated site I-associated transcripts processed by Rrp6 to M^∗^ intermediates, which in turn are trimmed to mature species, also by Rrp6. The turnover of II-pA RNAs is much slower and is not carried out by Rrp6 alone, except for the final digestion. In the *rrp6Δ* mutant, semimature snoRNAs are produced from II-pA precursors independently of Rrp6, possibly by the core nuclear exosome (see below). I^∗^ and I-pA RNAs are synthesized but remain dead-end products that are not chased into mature snoRNAs. Digestion of adenines is not carried out by the CCR4-NOT and Pan2-Pan3 deadenylase complexes, as *caf1Δ/ccr4Δ* and *ccr4Δ/pan2Δ* mutants do not show any accumulation of poly(A)^+^ snoRNAs (Figure S4), designating the nuclear exosome with the specific involvement of Rrp6 for this function.

### Contribution of Trf4/5 Polymerases to snoRNA Polyadenylation

**Figure 3:**
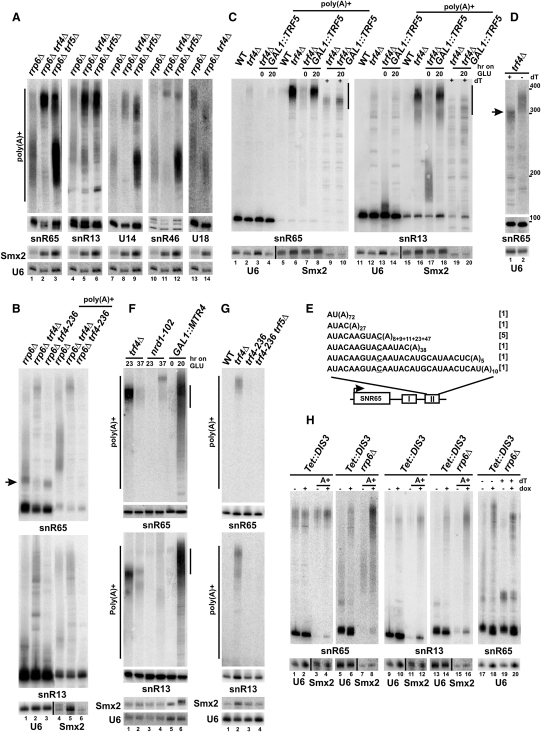
Polyadenylation and Processing of snoRNAs in TRAMP and Exosome Mutants (A–D and F–H) Northern analysis of polyadenylated pre-snoRNAs in total and poly(A)^+^ fractions from various strains. U6 and *SMX2* mRNA are loading controls for total and poly(A)^+^ RNAs, respectively. Site I species characteristic for *rrp6Δ* are indicated with an arrow in (B). Poly(A)^+^ fractions ([C], lanes 9 and 10, 19 and 20 and [H], lanes 19 and 20) or total RNA (D) deadenylated by RNase H treatment in the presence of oligo(dT). An arrow in (D) points at deadenylated pre-snR65 from terminator II; RNA size marker is on the right. To deplete Trf5 (C) or Mtr4 (F), *trf4Δ/GAL1::TRF5* or *GAL1::MTR4* cells were pregrown on YPGal and transferred to YPD; *trf4Δ* and *nrd1-102* cells were grown at 23°C or transferred to 37°C (F); Dis3 was depleted by growth in the presence of doxycycline (H). (E) CR-RT-PCR of polyadenylated pre-snR65 terminated in region II in the *trf4Δ* strain. The number of added A residues is in subscript.

Trf4/5 polymerases polyadenylate Rrp6-dependent RNA targets, including snoRNA Rnt1 degradation and processing intermediates (Egecioglu et al., 2006). Polyadenylation status of box C/D snR65, snR13, and U14 and box H/ACA snR46 not processed by Rnt1 was therefore analyzed in *rrp6Δ* strains lacking Trf4 or Trf5 (Figure 3A). High-mobility RNAs, which are strongly enriched in the poly(A)^+^ fraction and probably end at terminator II, visibly accumulate in *rrp6Δ/trf4Δ* and *rrp6Δ/trf5Δ* mutants, whereas site I polyadenylated species decrease in *rrp6Δ/trf4Δ* cells. Note that this phenotype is observed for both snoRNA classes. In contrast, poly(A)^+^ forms of pre-U18, generated by lariat debranching or Rnt1 cleavage, are not equally affected by deletion of *TRF4*, confirming that polyadenylation of other precursors is related to their termination. When deletion of *RRP6* is combined with a *trf4-236* catalytic site mutant with the abolished poly(A) polymerase activity (Wyers et al., 2005), both polyadenylated populations are missing, while a *trf4Δ-*like ladder of poly(A)^−^ intermediates and major site I precursors accumulate (Figure 3B). These short species migrate faster in the *rrp6Δ* strain with a *trf4-236* allele than with the wild-type *TRF4*, suggesting that they may lack oligo(A) tails. These results are consistent with participation of Trf4, possibly by oligoadenylation, in the poly(A) tail synthesis of site I transcripts and, when Trf4 is missing, by shifting termination to site II that is polyadenylated by another polymerase. Weaker effects in cells lacking Trf5 point to a minor role of this protein.

Similar accumulation of longer poly(A)^+^ pre-snoRNAs was observed in *trf4Δ* cells (Figure 3C). Since deletion of both *TRF4* and *TRF5* is lethal, Trf5 was depleted in the *trf4Δ/GAL1::TRF5* strain by the transfer to media containing glucose. Polyadenylation of snR65 and snR13 precursors at terminator II persists following depletion, showing that Trf4 and Trf5 polymerases are not, either individually or in a redundant fashion, required for the synthesis of this poly(A) tail. Some accumulation of site I poly(A)^+^ species is visible for snR13 in *trf4Δ/GAL1::TRF5* grown on galactose, where overexpression of Trf5 partially rescues *trf4Δ* phenotypes (Houseley and Tollervey, 2006; LaCava et al., 2005). RNaseH treatment of snR65 in the presence of oligo(dT) shows that in *trf4Δ* cells poly(A) tail of the average length of 70 nt starts approximately 200 nt from the mature 3′ end (Figure 3D). The location of poly(A) sites at various positions in terminator II of pre-snR65, that stretches over 50 nt, was confirmed by RT-PCR and CR-RT-PCR (Figure 3E and data not shown). The major site is shifted downstream by 20 nt compared with that in *rrp6Δ* (see Figure 1E), and it overlaps with the site directed by snR65 terminator within mRNA 3′UTR (underlined C in Figure 3F) (Steinmetz et al., 2006).

Effects observed in TRAMP mutants, i.e., following depletion of Mtr4 in the *GAL1::MTR4* strain (Figure 3F), in the strain devoid of both Air1/2 proteins and in a temperature-sensitive *trf4-ts896/trf5Δ* mutant (Figure S5), resemble the *trf4Δ* phenotype. This argues that TRAMP complex affects polyadenylation at terminators with Trf4 as a principal enzyme, given that additional inactivation of Trf5 has little input. In contrast, catalytic site mutants *trf4-236* and *trf4-236/trf5Δ* do not show accumulation of site II poly(A)^+^ precursors (Figure 3G), strongly suggesting that it is above all the absence of Trf4 that shifts polyadenylation toward this region. From these analyses we conclude that Trf4/5 polymerases are not involved in the addition of poly(A) tails at terminator II but may well act at site I. Trf4 may also contribute to the synthesis of major precursors independently of its polymerase activity.

To address the relative involvement of the core exosome versus its nuclear component, Rrp6, in the processing of polyadenylated precursors, exonuclease Dis3/Rrp44 expressed under the control of a Tet-regulated promoter (Dziembowski et al., 2007) was depleted in *Tet::DIS3* and *Tet::DIS3/rrp6Δ* strains by addition of doxycycline. The level of poly(A)^+^ snR65 and snR13 precursors from site II, but not from site I, is elevated already before depletion of Dis3 in *Tet::DIS3* cells and strongly increased after depletion in both strains (Figure 3H). Similar effects were observed for temperature-sensitive *rrp4-1* and *mtr3-1* mutants (Mitchell et al., 2003; van Hoof et al., 2000; Vasiljeva and Buratowski, 2006), indicating that the core exosome participates in the processing of only site II precursors. These phenotypes, and the appearance of extended nonpolyadenylated bands, match the traits of TRAMP mutants (Figure 3 and Figure S5; LaCava et al., 2005). Thus, as reported for other processes, the core exosome and Rrp6 have distinct impacts on accumulation of polyadenylated pre-snoRNAs. It appears that transcripts generated from terminator II are digested by the core exosome together with Rrp6, while site I precursors are matured entirely by Rrp6, in agreement with the pulse-stop analysis.

### Pre-snoRNAs Are Mainly Polyadenylated by Pap1

**Figure 4:**
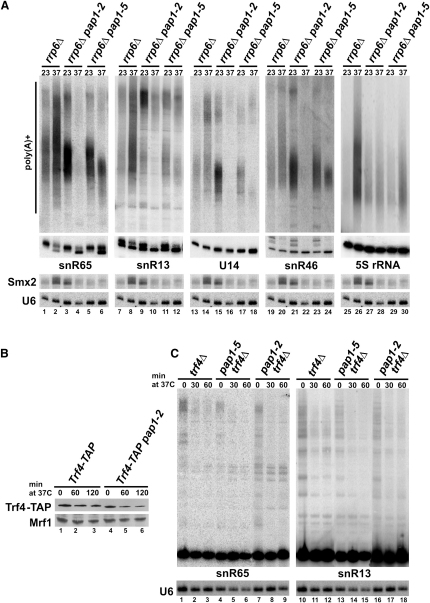
Major Poly(A) Polymerase Pap1p Is Involved in Polyadenylation of snoRNA Precursors (A) Northern analysis of polyadenylated pre-snoRNAs and 5S in *rrp6Δ*, *rrp6Δ/pap1-2*, and *rrp6Δ/pap1-5* strains grown at 23°C or transferred to 37°C for total (lower panel, mature RNAs) and poly(A)^+^ fractions (upper panel). (B) Western blot of Trf4-TAP in *Trf4-TAP* and *Trf4-TAP*/*pap1-2* strains grown at 23°C or transferred to 37°C. Trf4-TAP and Mrf1 (loading control) detected by peroxidase-anti-peroxidase or protein-specific antibodies, respectively. (C) SnR65 and snR13 in *trf4Δ/pap1-2* and *trf4Δ/pap1-5* strains grown at 23°C or shifted to 37°C.

Inactivation of Pap1 was shown to reduce poly(A)^+^ pre-snoRNAs in the *rrp6Δ* strain (van Hoof et al., 2000). To verify that it may participate in their polyadenylation, the pattern of poly(A)^+^ species was analyzed for snR65, snR13, and U14 in *rrp6Δ/pap1-2* and *rrp6Δ/pap1-5* strains (Figure 4A). As observed previously (Mitchell et al., 2003), some fully matured snoRNAs appear in the absence of Rrp6, mainly at 37°C, implying that Rrp6 is not a sole enzyme responsible for their final trimming. Both polyadenylated snoRNA forms are present in the poly(A)^+^ fraction for all strains in permissive conditions (23°C) but are hardly detectable in the *rrp6Δ/pap1-2* mutant and significantly decreased and shortened in *rrp6Δ/pap1-5* cells after transfer to 37°C. Note that *pap1-5* cells retain partial polyadenylation activity in nonpermissive conditions (Milligan et al., 2005). In contrast, poly(A) tails of 5S rRNA and some CUTs generated mainly by Trf4 endure Pap1 inactivation (Figure 4A; Wyers et al., 2005). Phenotypes in *pap1* mutants are therefore not an indirect result of depleting mRNAs. Specifically, the amount of Trf4 is not significantly altered by the *pap1-2* mutation (Figure 4B).

To confirm that Pap1 is involved in snoRNA polyadenylation in the absence of Trf4, double *trf4Δ/pap1-2* (Houseley et al., 2007) and *trf4Δ/pap1-5* strains were tested (Figure 4C). Poly(A)^+^ species are generally less abundant at 37°C in wild-type and some mutants, including *trf4Δ* (Figure 4C; Houseley et al., 2007; Milligan et al., 2005). At the permissive temperature, double mutants resemble *trf4Δ*, whereas poly(A) tails are significantly reduced after 30 min and virtually gone by 60 min following the shift to 37°C. These data provide further evidence that Pap1 participates in polyadenylation of pre-snoRNAs, particularly following termination at site II. Oligo(A) tails at 3′ ends of semimature snoRNAs in *rrp6Δ* cells are also not dependent on Trf4 or Trf5, as adenosines persist in *rrp6Δ/trf4Δ* or *rrp6Δ/trf5Δ* strains (Figure 1G and data not shown). These As most likely remain from poly(A) tails added by Pap1, since in the absence of Rrp6 snoRNAs are generated preferably from Pap1-dependent site II precursors.

### Polyadenylation of snoRNA Precursors Is Linked to Transcription Termination

**Figure 5:**
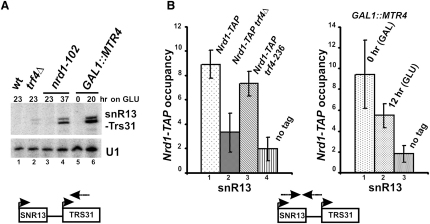
Components of Nrd1/Nab3 and TRAMP Complexes Cooperate in Termination at Site I (A) Readthrough transcription of the *SNR13* gene in *trf4Δ*, *nrd1-102*, and *GAL1::MTR4* mutants by primer extension from the downstream *TRS31* gene. U1 is used as a control. (B) Cotranscriptional association of Nrd1 with the *SNR13* gene by ChIP in different strains carrying *Nrd1-TAP*. *GAL1::MTR4* cells were pregrown on YPGal and transferred to YPD. Immunoprecipitated chromatin was amplified by qPCR using primers against the 3′ end of snR13 shown in the diagram below. ChIP signals were normalized by a background control from a nontranscribed region on chromosome V and corrected for Pol II occupancy along *SNR13* using Pol II CTD-specific antibody (8WG16). Error bars reflect standard deviation of four experiments.

In termination-deficient mutants, Pol II reads through snoRNA termination signals running into downstream genes. When TRAMP components are absent (*trf4Δ*) or depleted (*GAL1::MTR4*), readthrough of *SNR13* is detected by primer extension from the downstream *TRS31* gene, though it is weaker than for the *nrd1-102* mutant (Figure 5A). In addition, *nrd1-102*, *trf4Δ* and *GAL1::MTR4* strains show similar accumulation of transcripts polyadenylated at site II (Figure 3F and Figure S6).

These results suggest that TRAMP may affect snoRNA termination at site I. To address this question, cotranscriptional recruitment of Nrd1 to the *SNR13* gene was tested in the absence of Trf4 or following depletion of Mtr4. Chromatin immunoprecipitation (ChIP) was carried out for TAP-tagged Nrd1 in wild-type, *trf4Δ*, *trf4-236*, and *GAL1::MTR4* strains (Figure 5B). Nrd1 ChIP values were corrected for Pol II occupancy along *SNR13* to account for differences in transcription rates (Figure S7). A 3-fold decrease in the Nrd1 signal at *SNR13* to the level hardly above the untagged control was observed in the *trf4Δ* strain compared with the wild-type. Similarly, 2-fold reduction occurred after 12 hr of Mtr4 depletion, when growth and transcription rates were not significantly affected. In contrast, Nrd1 ChIP signal was not altered in the catalytic *trf4-236* mutant. This strongly indicates that TRAMP components, but not the polyadenylation activity of Trf4, contribute to the efficient association of Nrd1 with the chromatin. As Nrd1 and Nab3 binding is cooperative, and only Nrd1 contains a CTD-interacting domain (Carroll et al., 2007), association of Nab3 is probably also affected. The *NRD1* mRNA is subject to exosome- and TRAMP- mediated autoregulation (Arigo et al., 2006a), but the level of Nrd1 protein is not altered in *trf4Δ* cells (Figure S8).

To conclude, similar phenotypes observed in termination and TRAMP mutants and reduced Nrd1 presence at snoRNA genes in the absence of TRAMP components argue that these two complexes closely cooperate in 3′ end formation of pre-snoRNAs.

### Polyadenylation Is Required for snoRNA Synthesis

**Figure 6:**
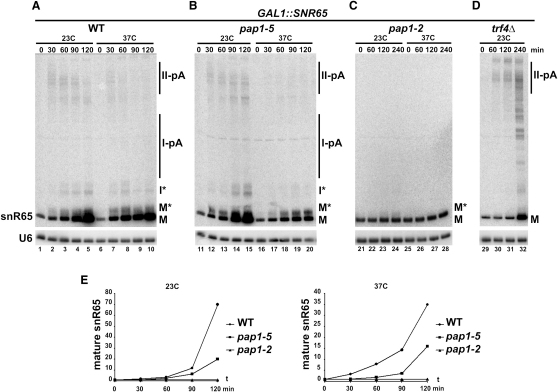
Pap1 and Trf4 Are Essential for snoRNA Synthesis Transcriptional pulse of snR65 in *GAL1::SNR65* (A), *GAL1::SNR65/pap1-5* (B), *GAL1::SNR65/pap1-2* (C), and *GAL1::SNR65/trf4Δ* (D) strains. Transcription of snR65 was induced for 120–240 min as indicated. Temperature-sensitive *pap1* cells were transferred to 37°C for 30 min before the pulse. RNA species are marked as in Figure 2. (E) PhosphorImager quantification of data from (A)–(C) for mature snR65. Values are standardized to the U6 control and expressed relative to levels before induction.

From the data presented so far, it appears that polyadenylation of pre-snoRNAs is related to termination. The most relevant question is whether it is the normal or secondary pathway of their synthesis. To address this, a transcriptional pulse of the inducible *GAL1::SNR65* was carried out in wild-type, *pap1-2*, *pap1-5*, and *trf4Δ* strains (Figure 6). SnR65 synthesis in wild-type and *pap1-5* cells at the permissive temperature proceeds as already described (see Figure 2A), via accumulation of polyadenylated II-pA and oligoadenylated I^∗^ precursors as well as Rrp6-dependent M^∗^ intermediates, followed by the buildup of the mature RNA. After transfer of *pap1-5* cells to 37°C for 30 min prior to the pulse, precursors derived from both terminators are markedly reduced and accumulation of mature species significantly inhibited (Figure 6E). The pulse in the *pap1-2* strain at both temperatures was very weak, yielding little, if any, polyadenylated precursors and no increase in mature snR65 (Figures 6C and 6E). The effects in *pap1* mutants are analogous to induction of hypoadenylated mRNAs (Hilleren et al., 2001; Milligan et al., 2005) and are not due to the inhibition of Pol II, as transcription is not affected (Birse et al., 1998; Ciais et al., 2008). These facts point to a direct role of Pap1 in the synthesis of snoRNAs. In the *trf4Δ* strain, II-pA species readily accumulate, but I^∗^ and M^∗^ are totally absent, which confirms that they derive from I-pA and not II-pA precursors. Despite the presence of polyadenylated precursors, mature snR65 is not produced efficiently: only after 4 hr of the pulse did the snoRNA level show a moderate increase. As transcription rates are similar in *trf4Δ* and wild-type cells (Figure S7), this outcome probably results from a slower, ineffective processing. The appearance of a characteristic ladder of nonpolyadenylated intermediates in the absence of Trf4 indicates that it may be involved in the synthesis of mature snoRNAs via rounds of adenylation followed by exonucleolytic trimming.

These data strongly suggest that polyadenylation by Pap1 and Trf4 polymerases linked to transcription termination is an intrinsic step in the snoRNA pathway that stimulates 3′ end formation by the exosome.

## Discussion

It has been puzzling for many years how two different kinds of RNAs, mRNAs that sport functionally important poly(A) tails and sn/snoRNAs devoid of such a striking feature, are produced by the same Pol II in yeast. Our work corroborates that snoRNA termination is carried out by two complexes and occurs in two regions: a major Nrd1/Nab3/Sen1-dependent terminator and a fail-safe terminator governed by the mRNA 3′ end formation machinery. More importantly, data presented here reveal that termination at both sites is followed by polyadenylation of precursors by Pap1 with contribution by Trf4 that is essential for efficient synthesis of mature snoRNAs and for RNA quality control. These findings also ultimately clarify that all Pol II transcripts in yeast, terminated by either mechanism, become polyadenylated by default and only the subsequent mode of the 3′ end processing defines the final form of the RNA.

### Which Poly(A) Polymerase?

Out of the two classes of poly(A) polymerases, Pap1 or Trf4/5, the latter option is consistent with observations that sn/snoRNAs polyadenylated in the *rrp6Δ* strain lose their poly(A) tail in the absence of Trf4 or Trf5 (Egecioglu et al., 2006; Houseley and Tollervey, 2006; LaCava et al., 2005). On the other hand, Pap1, too, was reported to polyadenylate several snoRNAs in *rrp6Δ* cells (van Hoof et al., 2000). Our data support both findings, but not in a wholly straightforward manner. Lack of Trf4 inhibits polyadenylation at site I, shifts termination toward the second region, and severely delays 3′ processing, while mutations in Pap1 abolish addition of poly(A) tails at both terminators and completely inhibit the synthesis of both precursor forms and mature snoRNAs. Thus, Pap1 appears to be a principal enzyme in this process.

Addition of poly(A) tails to transcripts terminated at the mRNA-like site by Pap1 is consistent with the well-established function of Pap1 as a component of the CPF in the context of cleavage and polyadenylation machinery. In wild-type cells, only a minority of precursors undergo termination through this site, but they are relatively stable, possibly through heterogeneous nuclear poly(A) RNA-binding proteins, Nab1 and Nab2 (Anderson et al., 1993; Wilson et al., 1994). The length of the poly(A) tail at terminator I (∼80 nt) and the analysis of *pap1* mutants point to the activity of Pap1 also at this site. We envisage that Trf4/5 may initiate the process by addition of short oligo(A) tails that are further extended by Pap1 to ensure effective recruitment of exonucleases. This hypothesis would explain how Pap1 recognizes substrates terminated by the Nrd1/Nab3 complex that has no apparent connection with Pap1 but interacts with Trf4. Similarly, both forms of *SRG1* CUT, of which the shorter *SRG1_S_* is generated by a Nrd1/Nab3-dependent termination, undergo Pap1-mediated polyadenylation (Thiebaut et al., 2006). Also, ncRNA IGS1-R from the intergenic rDNA spacer region is polyadenylated by Pap1 (Houseley et al., 2007; Vasiljeva et al., 2008a). This illustrates that transcripts produced via the Nrd1/Nab3 pathway can be Pap1 substrates.

### Cooperation between Nrd1/Nab3, TRAMP, and the Exosome

Nrd1/Nab3, the exosome, and TRAMP interact and function in termination and turnover of several RNAs, including numerous CUTs, snoRNAs, and their intermediates and even some mRNAs (Arigo et al., 2006a, 2006b; Egecioglu et al., 2006; Gudipati et al., 2008; Houseley et al., 2007; Thiebaut et al., 2006; Vasiljeva and Buratowski, 2006; Vasiljeva et al., 2008a; Wyers et al., 2005). The major role of Trf4 in these processes is to adenylate RNAs targeted for degradation, and binding of Nrd1/Nab3 has been suggested to stimulate recruitment of TRAMP/exosome to their substrates. Addition of adenines, however, is not the only function of Trf4. During snoRNA termination, lack of TRAMP components, Trf4 and Mtr4, affects the association of the Nrd1/Nab3 complex with snoRNA genes, contributing in this way to efficient termination. The mechanism underlying this connection is unclear, especially since Trf4 was not reported to interact with Pol II at snoRNA genes, and we also failed to detect such contacts (our unpublished data). It is more likely, then, that TRAMP secures Nrd1/Nab3 binding to nascent transcripts. Alternatively, as Mtr4 preferentially binds to poly(A) RNA (Bernstein et al., 2008), its helicase activity may be employed not only to recruit the exosome and facilitate its passage through RNA-protein structures (Houseley and Tollervey, 2006) but also to dissociate or remodel RNP complexes to release and recycle Nrd1/Nab3 following termination.

### Why Are snoRNAs Polyadenylated?

**Figure 7:**
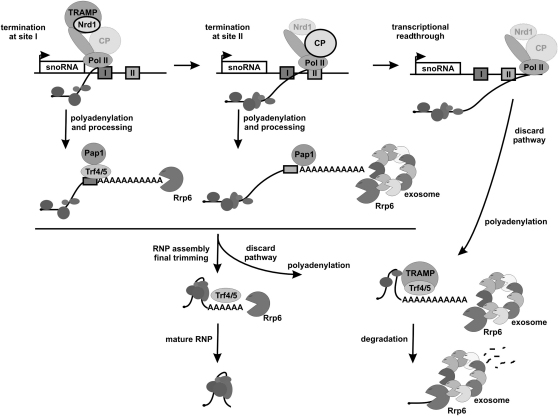
Model Correlating Termination, Polyadenylation, and Processing of snoRNAs The majority of snoRNAs terminate at site I in a Nrd1/Nab3-dependent pathway; the mRNA cleavage and polyadenylation complex, denoted CP and marked as semitransparent, is inactive. Released pre-snoRNAs are adenylated by Trf4/5, further polyadenylated by Pap1, and rapidly processed in subsequent rounds of adenylation by Trf4/5 and exonucleolytic digestion by Rrp6. Transcripts that had passed site I are terminated by components of CP at site II, while the Nrd1/Nab3 complex, marked as semitransparent, is released or inactive. Site II precursors are polyadenylated exclusively by Pap1 and processed by the core exosome/Rrp6. The last few nucleotides are trimmed by Rrp6. Readthrough transcripts and defective snoRNPs are degraded by the exosome/Rrp6 in the course of processing.

It has been established that poly(A) tail has a double, somehow antagonistic role in RNA stabilization and function and in RNA surveillance. Polyadenylation of snoRNAs, as well as other stable RNAs and CUTs, has been most often attributed to the mechanism of RNA quality control, though it has also been suggested to contribute to snoRNA processing (Egecioglu et al., 2006; van Hoof et al., 2000). Our data allow us to link these functions and show unambiguously that the poly(A) tail is required for efficient 3′ end processing of snoRNA precursors by the exosome/Rrp6. We propose a model for this process, illustrated in Figure 7. Both Nrd1/Nab3/Sen1 and mRNA 3′ end formation complexes are recruited to the CTD of Pol II early during transcription initiation, as reported. Owing to the RNA-binding capacity of Nrd1 and Nab3, these proteins assisted by TRAMP recognize the first terminator cooperatively and with a high specificity. Termination at this site is followed by the synthesis of the poly(A) tail by Pap1 with the contribution of Trf4 and subsequently by a very rapid trimming by Rrp6. Some fraction of transcripts that escaped termination reaches the second site and is released by the mRNA 3′ end formation machinery. These precursors are undoubtedly polyadenylated by Pap1, and their maturation is carried out by the core exosome together with Rrp6. When approaching the mature snoRNA 3′ end, and more specifically bound RNP proteins, the progress of exonucleases is slowed down and Rrp6 takes over the digestion of final nucleotides, including extra adenines. Progression of the exosome through the RNA is facilitated by the adenylation activity of Trf4.

Where, then, is the place in our model for the connection between polyadenylation and RNA surveillance? It is still an unsolved riddle how TRAMP/exosome recognizes defective RNA molecules. In the case of hypomodified tRNAs, their abnormal structure contributes to this process (Kadaba et al., 2004, 2006). For snoRNAs, their functionality is reflected by correct RNP composition; defective molecules will not assemble into proper particles. Hence transpires the possible role of polyadenylation-driven processing that allows sufficient time for remodeling of pre-RNP to mature RNP and the recognition and destruction of damaged molecules. Only RNAs assembled into proper RNPs during maturation will have their 3′ ends accurately processed and will be protected against degradation by advancing exonucleases. This view is supported by observations for the mutant in the core boxC/D snoRNP protein Nop1, which affects snoRNP stability and leads to the defect in transcription termination (Morlando et al., 2004) and to the accumulation of oligoadenylated intermediates that failed to be trimmed to mature species by Rrp6 (this work).

Our model also accounts for the generation of two classes of precursors that are processed with different kinetics. The second terminator may not only act as a fail-safe signal for the escapees but also ensure a pool of precursors, pending the need for further mature molecules. If the required level of snoRNAs is synthesized, and RNP proteins are limiting, these precursors are directed to the discard pathway.

One remaining question is where these processes are localized within the nucleus. In the *rrp6Δ* mutant, snoRNAs and poly(A)^+^ RNAs accumulate in a discrete nucleolar domain distinct from the nucleolar body (NB) or in nucleoplasmic foci in *rrp6Δ/rna14-1* and *rrp6Δ/rna15-2* strains (Carneiro et al., 2007, 2008). The authors reason that these spots are not sites of transcription but “surveillance centers” where aberrant RNAs are degraded by the exosome. It is possible, however, that these foci contain polyadenylated snoRNA precursors whose processing is slowed down in the absence of Rrp6. The fact that nucleolar poly(A) domains depend on both Pap1 and Trf4 argues that this may be the case. SnoRNA processing involves passage not only through NB, which is probably the site of final maturation (cap hypermethylation and final 3′ trimming), but also through other regions within the nucleus and nucleolus. We envisage that “processing” and “surveillance” centers are closely related, if not identical.

## Experimental Procedures

### Yeast Strains and Media

Strains used in this work are listed in Table S1. Construction of strains and growth conditions are described in the Supplemental Data.

### RNA Methods

General RNA methods are described in the Supplemental Data. Polyadenylated RNAs were isolated using Poly(A) Purist Mag kit (Ambion). Deadenylation of RNA was performed as described (LaCava et al., 2005) using 20 μg of RNA that had been hybridized to 10 pM of oligo(dT)_20_. For RT-PCR, 1 μg of DNaseI-treated (Roche) total RNA was reverse transcribed with AMV (Promega) using 10 pM ADAPT-oligo(dT)_30_ or gene-specific primers. cDNA was diluted 10- to 100-fold and used in PCR reactions with ADAPT-oligo(dT)_30_ and a primer against the mature snoRNA. Resulting bands were excised from agarose gels and sequenced. CR-RT-PCR was carried out on total RNA circularized with T4 RNA ligase (NEB). 5′ end with the TMG cap of snR13 and snR3 was removed prior to circularization by RNaseH treatment in the presence of W274 or W277 oligonucleotides. Circular molecules were amplified by RT-PCR, and products were cloned into pGEM-T Easy (Promega) and sequenced. Oligonucleotides, probes, and primers are listed in Table S2.

### Chromatin Immunoprecipitation

ChIP was performed as described (Houseley et al., 2007) using IgG Sepharose 6 Fast Flow (GE Healthcare) to immunoprecipitate Nrd1-TAP and 8WG16 antibody against CTD of Pol II (Covance) bound to protein G Sepharose 6 Fast Flow to measure Pol II occupancy. Precipitated and input chromatin was amplified with SYBR Green JumpStart Taq ReadyMix (Sigma) and Lightcycler 480 (Roche). qPCR was performed in triplicate. Quantification of ChIP values is described in the Supplemental Data.

## Supplemental Data

Document S1. Supplemental Experimental Procedures, Two Tables, Supplemental References, and Eight Figures
