# Supplementary material for: Polyadenylation Linked to Transcription Termination Directs the Processing of snoRNA Precursors in Yeast
Source: Mol Cell. 2008 Oct 24;32(2-3):247–58. doi: 10.1016/j.molcel.2008.10.003 (PMC2593888; doi:10.1016/j.molcel.2008.10.003)
Supplement: Document S1. Supplemental Experimental Procedures, Two Tables, Supplemental References, and Eight Figures [file mmc1.pdf]

Molecular Cell, Volume *32*

Supplemental Data

Polyadenylation Linked to Transcription Termination Directs the Processing of snoRNA Precursors in Yeast

Pawel Grzechnik and Joanna Kufel

Supplemental experimental procedures

Strain construction and growth

The transformation procedure was as described (Gietz et al., 1992). Strains were generated by one-step PCR procedure (Longtine et al., 1998). To construct strains expressing snR65 from an inducible *GAL1* promoter at the *SNR65* locus (strains *GAL1::SNR65*, *GAL1::SNR65/rrp6Δ*, *GAL1::SNR65/trf4Δ* and *GAL1::SNR65/pap1-5*) the region of *GAL1* promoter was amplified by PCR using the pFA6a-His3Mx6-pGAL1 plasmid and primers W297 and W298. The C-terminal *pap1-2/Trf4-TAP* strain was constructed according to (Puig et al., 2001) using W301 and W302 primers. To obtain the double *rrp6Δ/trf4-236* mutant *RRP6* was disrupted in the FWY9 strain with a His3Mx6 from pFA6a-His3Mx6 plasmid using W307 and W308 primers. *TRF4* disruption in the *Nrd1-TAP* strain was carried out by replacing *TRF4* ORF with kanMX6 marker amplified from pFA6a-kanMx6-pGAL1 template (Longtine et al., 1998) using W299 and W300 primers. The *trf4-236-HA* strain was generated by exchanging the TAP tag and *K. lactis* TRP1 sequences in the *trf4-236::TAP::K.lactis TRP1* gene in the FWY9 strain with a HA- kanMx6 cassette amplified by PCR using the pFA6a-3HA-kanMx6 plasmid and primers W305 and W306. *Nrd1-TAP* was introduced into *trf4-236-HA* and *GAL1::MTR4* strains using a pBS1539 plasmid (Rigaut et al., 1999) and primers W303 and W304. Strains were grown at 23°C or 30°C either in YPD or YPGal medium (1% yeast extract, 2% Bacto-peptone, 2% glucose or 2% galactose, respectively) or in synthetic complete medium (0.67% yeast nitrogen base, 2% glucose or 2% galactose, supplemented with amino acids and nucleotide bases). Temperature-sensitive strains were grown at 23°C and transferred to 37°C for 2 hours. Transcriptional pulse was induced by addition of 2% galactose to yeast cultures pre-grown in SC medium containing 2% raffinose and 0.08% glucose. *Pap1-2* and *pap1-5* strains were transferred to 37°C for 30 min prior to the pulse. For pulse-stop experiments 4% glucose was added following the pulse. To deplete

Tfr5 or Mtr4 in *trf4Δ/GAL1::TRF5* or *GAL1::MTR4* and *Nrd1-TAP/GAL1::MTR4* strains, respectively, cells were transferred from YPGal to YPD medium for 12h (*Nrd1-TAP/GAL1::MTR4*) or 20h (*trf4Δ/GAL1::TRF5* and *GAL1::MTR4)*, whereas Dis3 in *Tet::DIS3* and *rrp6Δ/Tet::DIS3* strains was depleted by addition of doxycycline for 20h (2.5μg/ml).

General RNA methods

Total RNA from yeast cells was isolated using a hot phenol procedure (Schmitt et al., 1990). Northern hybridization and primer extension were essentially as described (Tollervey and Mattaj, 1987). Radioactive probes were either 5'-end γ-^{32}P-labelled oligoprobes or α-^{32}P internally labelled random-primed probe (for SmX2 mRNA) prepared using PCR product as template and DECAprime II Kit (Ambion). 8μg of total RNA or 0.5μg of poly(A)$^+$ RNA and 2μg of total RNA in the case of purified poly(A)$^+$ samples were separated on 6% denaturing polyacrylamide-urea gels, transferred onto nylon membranes and hybridized with oligonucleotide probes listed in supplementary Table S2.

Western blot analysis

Western blot analysis was performed using peroxidase-anti-peroxidase antibody to detect Nrd1-TAP and Trf4-TAP and polyclonal anti-Mrf1 antibody followed by horseradish peroxidase-conjugated goat anti-rabbit antibody.

Calculation of ChIP values

Nrd1-TAP occupancy at *SNR13* in *trf4Δ*, *trf4-236* and *GAL1::MTR4* (in nonpermissive conditions, growth in glucose) in Figure 5B was compared to the level in the otherwise wild-type Nrd1-TAP control or *Nrd1-TAP/GAL1::MTR4* cells (in permissive conditions, growth on galactose).

ChIP values for Nrd1-TAP were quantified using the formula $2^{-\Delta\Delta Ct} = 2^{-((Ct\ IP\ target\ gene\ -\ Ct\ Input\ target\ gene)\ -\ (Ct\ IP\ control\ -\ Ct\ Input\ control))}$, where "Ct IP" and "Ct Input target gene" are cycle numbers for the *SNR13* gene and "Ct IP" and "Ct Input control" are cycle numbers for non-coding region on chromosome V. ChIP values for Pol II were determined using $2^{-\Delta Ct} = 2^{-(ct\ IP\ -\ ct\ background)}$, where "Ct IP" is cycle number for immunoprecipitate and "Ct background" is cycle number for control without antibodies. ChIP levels for Nrd1-TAP in different strains were corrected for Pol II occupancy.

Table S1. Yeast strains used in this work.

strain	description	reference
BY4741	*MATa his3Δ1; leu2Δ0; met15Δ0; ura3Δ0*	Euroscarf
trf4Δ	as BY4741 but *TRF4::kanMX4*	Euroscarf
trf5Δ	as BY4741 but *TRF5::kanMX4*	Euroscarf
trf4Δ/GAL1::TRF5	as *trf4Δ* but *HisMX6-pGAL1-HA::TRF5*	(LaCava et al., 2005)
rrp6Δ	as BY4741 but *RRP6::kanMX4*	Euroscarf
rrp6Δ/trf4Δ	as *trf4*-Δ but *RRP6::natMX6*	(LaCava et al., 2005)
rrp6Δ/trf5Δ	as *trf5*-Δ but *RRP6::natMX6*	(LaCava et al., 2005)
W303	*MATa his3-11,15 trp1-1; leu2-3,112 ura3-1 ade2-1*	(Sikorski and Hieter, 1989)
CY1243 *trf4-836 trf5Δ*	as W303 but *trf4-ts896::HIS3 TRF5::LEU2*	(Wang et al., 2000)
BMA64	*MATa, ura3-1, ade2-1, his3-11,5, trp1Δ, leu2-3,112, can1-100*	(Baudin et al., 1993)
FWY9 *trf4-236*	as BMA64 but *trf4-236::TAP::K.lactis TRP1*	(Wyers et al., 2005)
FWY10 *trf4-236 trf5Δ*	as FWY9 but *trf5Δ::HIS3*	(Wyers et al., 2005)
air1Δ	as BY4741 but *AIR1::kanMX4*	Euroscarf
air2Δ	as BY4741 but *AIR2::kanMX4*	Euroscarf
air1Δ/air2Δ	as BY4741 but *AIR1::kanMX4 AIR2::natMX6*	(LaCava et al., 2005)
pap1-2	MATa *ade2 his3 trp1 ura3 leu2 LEU2::PAP1 pap1-2*	(Minvielle-Sebastia et al., 1994)
pap1-5	MATa *ade2 his3 trp1 ura3 leu2 LEU2::PAP1 pap1-5*	(Minvielle-Sebastia et al., 1994)
rrp6Δ/pap1-2	as *pap1-2* but *RRP6*:: *K.lactis URA3*	(Milligan et al., 2005)
rrp6Δ/pap1-5	as *pap1-5* but *RRP6*:: *K.lactis URA3*	(Milligan et al., 2005)

pap1-5/trf4Δ	as *pap1-5* but *TRF4::kanMX4*	this work
pap1-2/trf4Δ	as *pap1-2* but *TRF4::kanMX4*	(Houseley et al., 2007)
Trf4-TAP	as BY4741 but *TRF4::TAP::HIS3*	this work
Trf4-TAP/pap1-2	as *pap1-5 but TRF4::TAP::HIS3*	this work
GAL1::SNR65	as BY4741 but *GAL1::SNR65::HIS3*	this work
rrp6Δ/GAL1::SNR65	as *rrp6Δ* but *GAL1::SNR65::HIS3*	this work
pap1-5/GAL::SNR65	as *pap1-5* but *GAL1::SNR65::HIS3*	this work
trf4Δ̃GAL1::SNR65	as *trf4Δ* but *GAL1::SNR65::HIS3*	this work
YJL1166	*MATα ura3Δ0 his3Δ1 leu2Δ0 met15Δ0*	(Conrad et al., 2000)
YJL1163 *nrd1-102*	as YJL1166 but *nrd1-102*	(Conrad et al., 2000)
nrd1-5	as YJL1166 but *nrd1-5*	(Conrad et al., 2000)
rrp6Δ/nrd1-102	as *nrd1-102* but RRP6*::kanMX4*	(Houalla et al., 2006)
Nrd1-TAP	*MATa NRD1::TAP::HIS3MX6*	Open Biosystems
Nrd1-TAP/trf4Δ	as *Nrd1-TAP* but *TRF4::kanMX4*	this work
trf4-236-HA	as BMA64 but *trf4-236::HA::kanMX4*	this work
Nrd1-TAP/trf4-236	as *trf4-236-HA but NRD1::TAP::HIS3*	this work
rrp6Δ/trf4-236	as FWY9 but *RRP6::HISMX6*	this work
rrp6Δ/rna14-1	*MATa ade2-1 his3-11 leu2-3,112 trp1-1 ura3-1 rna14-1 RRP6:: K.lactis TRP1*	(Torchet et al., 2002)
rrp6Δ/rna15-1	*MATa ade2-1 his3-11 leu2-3,112 trp1-1 ura3-1 rna12-2 RRP6:: K.lactis TRP1*	(Torchet et al., 2002)
nop1-2	*ura3 leu2 nop1-2*::*HIS3*	(Tollervey et al., 1993)
Tet::Dis3	as W303 but *LYS2::DIS3 Tet::DIS3*	(Dziembowski et al., 2007)
Tet::Dis3 rrp6Δ	as *Tet::DIS3* but *RRP6::kanMX4*	(Dziembowski et al., 2007)
GAL1::MTR4	*MATα ade2-1 his3 leu2 trp1 ura3 HIS5sp-GAL1-3HA-MTR4*	(Torchet et al., 2002)
GAL1::MTR4/Nrd1-TAP	as *GAL1::MTR4* but *NRD1::TAP:: K.lactis URA3*	this work

Table S2. Oligonucletides used in this work.

primer	name	sequence
hybridization probes		
W035	snR13	CAACTCGAGCCAAATGCACTC
W036	snR33	CTTTCAATCTCTGCTCCTCC
W037	snR3	CAACTAGCAATCCACTCGAG
W038	snR43	TTCAAAGCTTGATCTTCTCC
W270	snR46	TTAGGCCTCGCTTTGAATCC
261	U6	AAAACGAAATAAATTCTTTGTAAAAC
205	U18	GTCAGATACTGTGATAGTC
202	U14	TCACTCAGACATCCTAGG
W076	snR68-2	AAGAGTCAATTTCCTCGGTA
W085	snR64-2	GATGTTCCTCGTCACTTGAG
W271	snR65	GCTTTCAGATACTATCTAGC
W045	snR50	CTGCTGCAAATTGCTACCTC
W309	5smx3	AGCGAGAGCAGTGATATCAG
W310	3smx3	TTAGTTCGGCAGCTCCCTG
primer extension		
262	U1	CAATGACTTCAATGAACAATTAT
W288	3snr65sp	ATAATCAAATCAGCTCATAC
W289	65RTligP	TACCAAGAGTTACAAAATC
W041	Trs31son	GTTGAATTATTCTTTGAGAC
RT-PCR		
W290	ADAPT-dT	CACTCGAGTTTTTTTTTTTTTTTTTTTTTTTTTTTT
W291	5snR65	TAAAATGATGATTTTTTTAAAC
W292	5snR13	AGGAAGTTTTTTCCTTTTTATATG
CR-RT-PCR		
W091	68RTlig	GATAACGCAGTAAAATAAATG
W092	68PCRlig	GTACAGTCTGTTTTATAATC
W272	50RTlig	AATCTGCTGCAAATTGCTAC
W273	50PCRlig	TGAATCAAACAAAGATTAAC
W274	13Hlig	AAAAAGGAAAAAACTTCCT
W275	13RTlig	ACAGCAACTCGAGCCAAATG

W276	13PCRlig	TTGCCAAATCAGTAACGGTG
W277	3Hlig	ATTAGTACTTTAGGACAAAG
W278	3RTlig	CGCTTATCACGAATAAGACC
W279	3PCRlig	CATTTATAAGAACTCGAGTG
W280	43RTlig	TATAGAACCCATGTCCCGTG
W281	43PCRlig	TTGATACAACCGTAGACGGC
W282	65RTlig	GTTAAGAAGATTCAAGATTGC
W283	65PCRlig	AGCTGATTTGATTATGGGCG
W284	65RTligD	GCTTTCAGATACTATCTAG
W285	65PCligD	TTATGATTACAGTGTTTTC
W286	U18ligRT	TTCCCATCATAAACACGGAC
W287	U18ligPC	GAGATGTGGTTGACTATCAC
ChIP		
W293	13-2up	CTGACCTTTTAACTTCCCCGTAG
W294	13-2low	CTGTCGCTTCCGTGTCTCTTGTCCTG
W295	5ChV	CTGTCAGAATATGGGGCCGTAG
W296	3ChV	CCATACCCTCGGGTCAAACAC
W304	5sn65	GCTTCACCAGATGTTCTTGTC
W304	3sn65	TACCAAGAGTTACAAAATCG
strain construction		
W297	5GLSNR65	TTTTATGCGCGCCTCCTTCAAAAAAAAATTTACATATATAAC ATAGGTGGGAATTCGAGCTCGTTTAAAC
W298	3GLSNR65	GTGGAAAAAAAAATGACGTTCTCGTCATTGTCATCTTCCAG GTCTACGCCTTTGAGATCCGGGTTTT
W299	5TRF4d	ATGGGGGCAAAGAGTGTAACAGCCTCTTCTTCAAAGAAGAT TAAAAACCGCGGATCCCCGGGTTAATTAA
W300	3TRF4d	TTAAAGGGTATAAGGATTATATCCATCTTCATCATCATCTTC GCTAGAGAGAATTCGAGCTCGTTTAAAC
W301	5Trf4TAP	CTGTCTCTAGCGAAGATGATGATGAAGATGGATATAATCCT TATACCCTTTCCATGGAAAAGAGAAG
W302	3Trf4TAP	CAGAAGTAAAATTAAATTCAATTATTAATACATAATACGCA TATTTAAACTACGACTCACTATAGGG
W303	5TAPnrd1	ATTCTTTGATGAATATGCTTAACCAACAGCAGCAGCAACAA

		CAACAAAGCTCCATGGAAAAGAGAAG
W304	3TAPnrd1	GAACATAGGAAAAAACAGAAATTATATATAGAGGTAGATT AGTTTTATGTTACGACTCACTATAGGG
W305	5HAtrf4fa	CTGTCTCTAGCGAAGATGATGATGAAGATGGATATAATCCT TATACCCTTCGGATCCCCGGGTTAATTAA
W306	3TAPmycfa	CATGATTGCATGGTATCACTACACACATCCCATATACCCCG GTATCTCTCGAATTCGAGCTCGTTTAAAC
W307	5RRP6d	TAGACGAAATAGGAACAACAAACAGCTTATAAGCACCCAA TAAGTGCGTTCGGATCCCCGGGTTAATTAA
W308	3RRP6d	ATGAAAATTACCATAATTTATAAATAAAAAAATACGCTTGT TTTACATAAGAATTCGAGCTCGTTTAAAC

Supplemental References

Baudin, A., Ozier-Kalogeropoulos, O., Denouel, A., Lacroute, F., and Cullin, C. (1993). A simple and efficient method for direct gene deletion in *Saccharomyces cerevisiae*. Nucleic Acids Res. *21*, 3329–3330.

Conrad, N. K., Wilson, S. M., Steinmetz, E. J., Patturajan, M., Brow, D. A., Swanson, M. S., and Corden, J. L. (2000). A yeast heterogeneous nuclear ribonucleoprotein complex associated with RNA polymerase II. Genetics *154*, 557-571.

Dziembowski, A., Lorentzen, E., Conti, E., and Seraphin, B. (2007). A single subunit, Dis3, is essentially responsible for yeast exosome core activity. Nat. Struct. Mol. Biol. *14*, 15-22.

Gietz, D., St Jean, A., Woods, R. A., and Schiestl, R. H. (1992). Improved method for high efficient transformation of intact yeast cells. Nucleic Acids Res. *20*, 1425.

Houalla, R., Devaux, F., Fatica, A., Kufel, J., Barrass, D., Torchet, C., and Tollervey, D. (2006). Microarray detection of novel nuclear RNA substrates for the exosome. Yeast *23*, 439-454.

Houseley, J., Kotovic, K., El Hage, A., and Tollervey, D. (2007). Trf4 targets ncRNAs from telomeric and rDNA spacer regions and functions in rDNA copy number control. EMBO J. *20*, 4996–5006.

LaCava, J., Houseley, J., Saveanu, C., Petfalski, E., Thompson, E., Jacquier, A., and Tollervey, D. (2005). RNA degradation by the exosome is promoted by a nuclear polyadenylation complex. Cell *121*, 713-724.

Longtine, M. S., McKenzie, A. r., Demarini, D. J., Shah, N. G., Wach, A., Brachat, A., Philippsen, P., and Pringle, J. R. (1998). Aditional modules for versatile and economical PCR-based gene deletion and modification in *Saccharomyces cerevisiae*. Yeast *14*, 953-961.

Milligan, L., Torchet, C., Allmang, C., Shipman, T., and Tollervey, D. (2005). A nuclear surveillance pathway for mRNAs with defective polyadenylation. Mol. Cell. Biol. *25*, 9996-10004.

Minvielle-Sebastia, L., Preker, P. J., and Keller, W. (1994). RNA14 and RNA15 proteins as components of a yeast pre-mRNA 3'-end processing factor. Science *266*, 1702-1705.

Puig, O., Caspary, F., Rigaut, G., Rutz, B., Bouveret, E., Bragado-Nilsson, E., Wilm, M., and Séraphin, B. (2001). The tandem affinity purification (TAP) method: a general procedure of protein complex purification. Methods *24*, 218-229.

Rigaut, G., Shevchenko, A., Rutz, B., Wilm, M., Mann, M., and Seraphin, B. (1999). A generic protein purification method for protein complex characterization and proteome exploration. Nat. Biotechnol. *17*, 1030-1032

Schmitt, M. E., Brown, T. A., and Trumpower, B. L. (1990) A rapid and simple method for preparation of RNA from Saccharomyces cerevisiae. Nucleic Acids Res. *18*, 3091-3092.

Sikorski, R.S. and Hieter, P. (1989) A system of shuttle vectors and yeast host strains designed for efficient manipulation of DNA in Saccharomyces cerevisiae. Genetics, *122*, 19–27.

Tollervey, D., Lehtonen, H., Jansen, R., Kern, H., and Hurt, E. C. (1993). Temperature-sensitive mutations demonstrate roles for yeast fibrillarin in pre-rRNA processing, pre-rRNA methylation, and ribosome assembly. Cell *72*, 443-457.

Tollervey, D., and Mattaj, I. W. (1987) Fungal small nuclear ribonucleoproteins share properties with plant and vertebrate U-snRNPs. EMBO J. *6*, 469-476.

Torchet, C., Bousquet-Antonelli, C., Milligan, L., Thompson, E., Kufel, J., and Tollervey, D. (2002). Processing of 3' extended read-through transcripts by the exosome can generate functional mRNAs. Mol. Cell *9*, 1285-1296.

Wang, Z., Castano, I. B., De Las Penas, A., Adams, C., and Christman, M. F. (2000). Pol kappa: A DNA polymerase required for sister chromatid cohesion. Science *289*, 774-779.

Wyers, F., Rougemaille, M., Badis, G., Rousselle, J. C., Dufour, M. E., Boulay, J., Regnault, B., Devaux, F., Namane, A., Seraphin, B., *et al.* (2005). Cryptic pol II transcripts are degraded by a nuclear quality control pathway involving a new poly(A) polymerase. Cell *121*, 725-737.

S1.

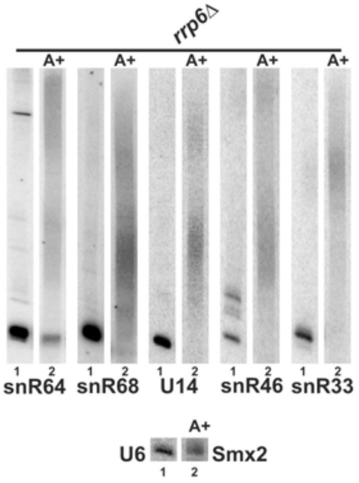

S4.

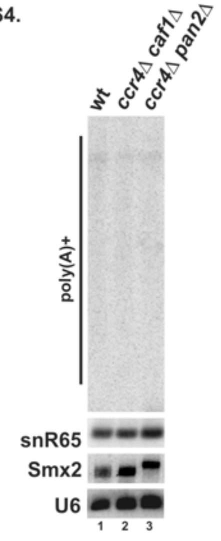

S5.

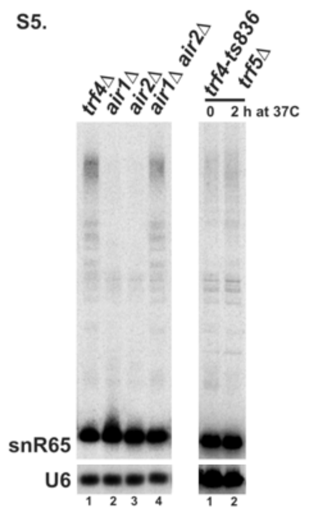

S2.

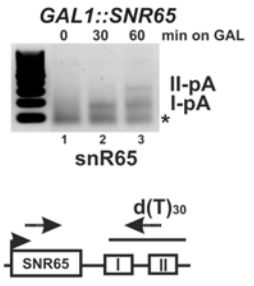

S3.

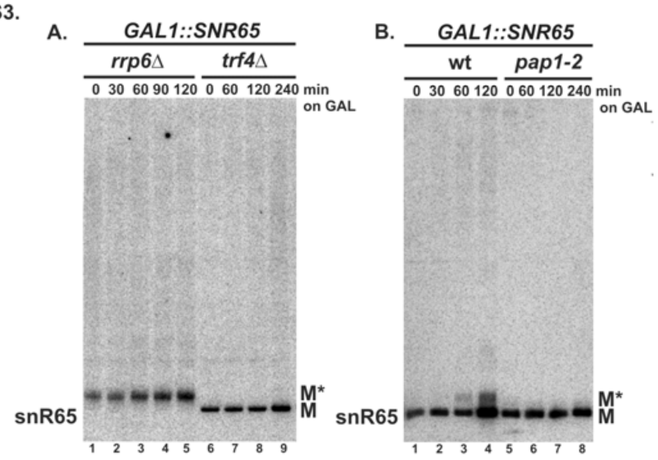

S6.

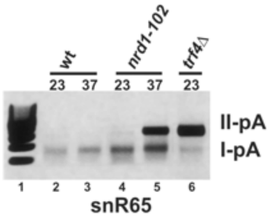

S7.

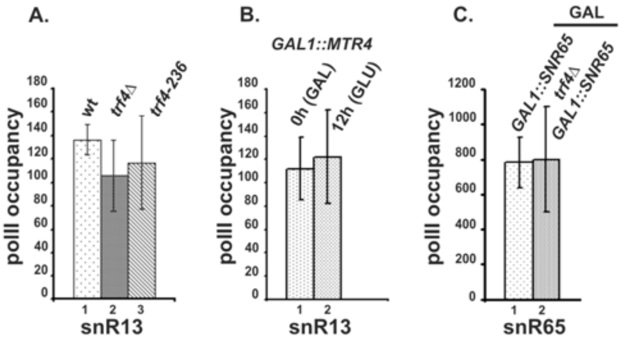

S8.

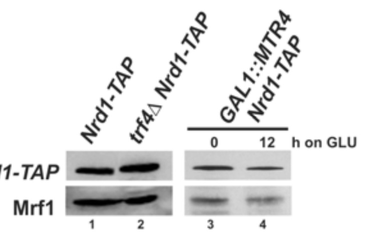

Figure S1.

Box C/D and box H/ACA snoRNA precursors are polyadenylated in the *rrp6Δ* strain. Northern hybridization of total and poly(A)$^+$ RNA (lane A$^+$) from the *rrp6Δ* strain probed for box C/D (snR64, snR68, U14) and box H/ACA (snR46 and snR33) snoRNAs. U6 (lane 1) and *SMX2* mRNA (lane 2) are used as loading controls for total and poly(A)$^+$ RNAs, respectively.

Figure S2

Both I-pA and II-pA precursors are detected during the transcriptional pulse in wild-type cells.

RT-PCR analysis of polyadenylated pre-snR65 in *GAL1::SNR65* strain where transcription was induced by addition of galactose for times indicated. Reverse transcription was performed using using ADAPT-oligo(dT)$_{30}$ and cDNA was amplified using ADAPT-oligo(dT)$_{30}$ and a primer specific for mature snR65. The asterisk indicates primer-dimers.

Figure S3

Transcriptional pulse of snR65 under the control of the *GAL1* promoter generates mature (M) snoRNA in wild-type, *trf4Δ, pap1-2* strains but only untrimmed semi-mature (M*) species in *rrp6Δ* cells. Cells were grown at 23˚C in SC medium (2% raffinose, 0.08% glucose) and transcription of snR65 was induced for times indicated by addition of galactose. I-pA and II-pA, polyadenylated precursors from respective termination sites; I*, oligoadenylated precursor from site I; M*, semi-mature species; M, mature snoRNA.

Figure S4

Deadenylases are not involved in removal of poly(A) tails.

Northern analysis of snR65 in *ccr4Δ/caf1Δ* and *ccr4Δ/pan2Δ* strains. Total RNA (lower panel with mature snoRNA) and the poly(A)$^+$ fraction (upper panel). U6 and SmX2 mRNA, loading controls for total and poly(A)$^+$ RNAs, respectively.

Figure S5

TRAMP components Air1/2 are also involved in snoRNA processing. Northern analysis of polyadenylated snR65 in *trf4Δ*, *air1Δ, air2Δ*, *air1Δ/air2Δ* and *trf4-ts836/trf5Δ* strains. Only deletion of both Air proteins result in the phenotype comparable to that in the *trf4Δ* strain as

had been observed for several effects characteristic for TRAMP mutants (LaCava et al., 2005; Wyers et al., 2005). U6 is used as a control.

Figure S6

Mutation in Nrd1 and lack of Trf4 shift polyadenylated snR65 precursors towards site II. RT-PCR analysis of polyadenylated pre-snR65 in wild-type, *nrd1-102* and *trf4Δ* strains grown at 23˚C or shifted to 37˚C for 2 hours. Reverse transcription was performed using using ADAPT-oligo(dT)$_{30}$ and the cDNA was amplified using ADAPT-oligo(dT)$_{30}$ and a primer specific for mature snR65.

Figure S7

Transcription rate of endogenous snR13 is not altered in the absence of Trf4 and by the *trf4-236* mutation (A) or following depletion of Mtr4 (B). SnR65 under the control of *GAL1* promoter is transcribed with similar rates in wild-type and *trf4Δ* strains (C).
Pol II occupancy along *SNR13* or *SNR65* was analysed by ChIP using 8WG16 antibodies against the CTD of Pol II in *Nrd1-TAP* (wt), *Nrd1-TAP*/*trf4Δ (trf4Δ)*, *Nrd1-TAP*/*trf4-236 (trf4-236)* strains (A), in *Nrd1-TAP*/*GAL1::MTR4* (*GAL1::MTR4*) cells before (GAL) and after (GLU, 12 hours) depletion (B) and in *GAL1::SNR65, trf4Δ/GAL1::SNR65* cells grown in GAL (C). Error bars reflect standard deviation of three experiments.

Figure S8

Deletion of Trf4 and depletion of Mtr4 do not affect the level of Nrd1 protein.
Western blot of Nrd1-TAP, detected with peroxidase-anti-peroxidase antibodies, in *Nrd1-TAP, Nrd1-TAP*/*trf4Δ* and *Nrd1-TAP*/*GAL1::MTR4* strains. Mrf1 protein detected with protein-specific antibodies was used as a loading control.
